# The Predictive Value of the Syntax Score in Patients With Chronic Coronary Artery Disease Undergoing Percutaneous Coronary Intervention or Coronary Artery Bypass Grafting: A Pilot Study

**DOI:** 10.2174/1874192401711010028

**Published:** 2017-04-17

**Authors:** K. Papadopoulos, I. Lekakis, E. Nicolaides

**Affiliations:** 1 Cardiology Department, Medical Check-up Centre, 21^st^ Costa Anaxagora Street, 2014, Nicosia, Cyprus.; 2 Cardiology Department, Attikon University Hospital, Rimini 1, Chaidari 124 62, Athens, Greece.; 3 Saint George’s Medical School, University of Nicosia Medical School, 46 Makedonitissas Avenue, Engomi, 1700, Nicosia, Cyprus.

**Keywords:** Coronary artery disease, Percutaneous coronary intervention, Coronary artery bypass grafting, Syntax score, Risk assessment, American Heart Association (AHA)

## Abstract

**Objectives:**

To evaluate the usefulness of the SYNTAX score (SS) in predicting 1-year clinical outcomes in a population of patients with chronic coronary artery disease (CAD) undergoing percutaneous coronary intervention (PCI) or coronary artery bypass grafting (CABG).

**Background:**

Despite the proven prognostic value of the SS in patients with multivessel and/or left main (LM) CAD, its usefulness in other patient subsets remains uncertain.

**Methods:**

This was a prospective single centre cohort study conducted from September 2012 to November 2014 at the Nicosia General Hospital, Cyprus. Patients (n=140*;* 94% men and 6% women) with chronic CAD undergoing revascularization with either PCI or CABG were evaluated.

**Results:**

At 1-year, angina occurred in 20 patients (14.3%), myocardial infarction (MI) in 3 patients (2.1%), repeat revascularization procedures in 9 patients (6.4%) and death in 12 patients (8.6%). The SS independently predicted angina (p=0.024) but was not predictive of MI (p=0.964), death (p=0.292) or repeat revascularization (p=0.069).

**Conclusion:**

In this patient population, the SS predicted angina in the year following revascularization but was not predictive of MI, death or repeat revascularization.

## INTRODUCTION

The SYNTAX score (SS) is an angiographic tool to help cardiologists, interventionalists and surgeons grade coronary artery lesion complexity. The score represents a combination of the American Heart Association (AHA) classification of coronary tree segments modified for the Arterial Revascularization Therapies Study (ARTS) study, a modified Leaman score, the American College of Cardiology (ACC)/AHA lesion classification system, a combination of the Duke and Institut Cardiovasculaire Paris Sud (ICPS) classification system for bifurcation lesions, a chronic total occlusion classification system and “consultation with experts” [1, 2].

The SS was developed to quantify lesion complexity. It has also been used to assess prognosis in patients with multivessel coronary artery disease (CAD) and/or those with left main (LM) coronary artery lesions [3-14]. More recent data have shown that the SS predicts peri-procedural myocardial infarction (MI) in patients undergoing elective percutaneous coronary intervention (PCI) [15].

Despite its proven prognostic value in patients with multivessel and/or left main coronary artery disease (LM CAD), its usefulness in other subsets of patients remains uncertain. Here we aimed to evaluate the usefulness of the SS to predict 1-year clinical outcomes in a population of patients with chronic CAD (1 or 2 vessel, multivessel and/or LM involvement) undergoing PCI or coronary artery bypass grafting (CABG).

## MATERIAL AND METHODS

### Patient Population

This was a prospective single centre cohort pilot study conducted from September 2012 to November 2014 at the Nicosia General Hospital, Cyprus. Patients (n=140*;* 94% men and 6% women) with chronic CAD undergoing revascularization with either PCI or CABG were evaluated. The diagnosis of chronic CAD was based on the presence of symptoms of stable angina or a positive for myocardial ischemia stress test (exercise tolerance test, stress ECHO, or thallium scintigraphy). Patients presenting with unstable angina, non-ST elevation MI (non-STEMI), and ST elevation MI (STEMI) were excluded. Patients undergoing CABG for valve surgery were also excluded. The National Ethics Committee of Cyprus approved the study. Written informed consent was obtained from all patients and controls according to committee guidelines.

### Calculation of the SS

Seventy patients were treated with PCI and seventy with CABG. An experienced cardiologist calculated the SSs based on angiograms. Each coronary lesion with stenosis ≥50% of a vessel of ≥1.5 mm in diameter was separately scored using the SS score algorithm. Individual scores were summed to provide the overall SS. The patients were divided into tertiles according to the SS *:* lower SS tertile (SS ≤22), intermediate SS tertile (SS 23 to 32) and higher SS tertile (SS ≥32). Characteristics such as sex, age, and risk factors for CAD (Table **1**) were also recorded. Diabetes Mellitus was defined as a medical history of physician-diagnosed diabetes, overweight as a BMI between 25-30 and family history as a family history of premature CAD (<55 years in men and <65 years in women, first degree relatives). CAD was defined as a medical history of physician-diagnosed CAD.

### Associations Between SS and Clinical Outcomes

Major adverse cardiac events (MACE) including cardiac death, non-fatal acute MI, angina, and repeat revascularization of the target vessel over 1 year of follow-up were considered as the primary outcome. All deaths were considered cardiac unless another cause was definitively established. Acute MI was confirmed by evidence of 3-fold or greater creatine kinase-MB fraction elevation with symptoms or electrocardiographic evidence of myocardial ischemia. Recurrent angina was defined as the occurrence of chest pain due to myocardial ischaemia. Repeat revascularization included repeat PCI or CABG.

Telephone surveys were used to collect data for all patients. The survey took place 1 year after treatment and information on 4 outcomes was recorded*:* angina, MI, target lesion revascularization, and death. The answers were in the binary form of “yes” or “no”.

### Statistical Analysis

Data were analysed using SPSS 22 (IBM Statistics, Chicago, IL) and logistic regression was used to establish whether the SS was a significant predictor of the 4 outcomes.

## RESULTS

At 1-year follow up, angina occurred in 20 patients (14.3%), MI in 3 patients (2.1%), repeat revascularization procedures in 9 patients (6.4%) and death in 12 patients (8.6%) (Table **2**).

A logistic regression analysis was performed to ascertain the effects of SS, total cholesterol, HDL-C (high-density lipoprotein), LDL-C (low-density lipoprotein), triglycerides, age, sex, smoking, hyperlipidaemia, hypertension, diabetes mellitus, family history and overweight on the likelihood of the occurrence of angina, MI, target lesion revascularization and death, 1 year after the treatment.

Applying logistic regression with angina as the dependent variable, the SS was the only clinicopathological variable to significantly predict angina occurrence with an odds ratio of 0.948 (p=0.024) (Table **3**). The model explained 31.9% (Nagelkerke R^2^) of the variance in angina occurrence and correctly classified 87.9% of cases in the data.

However, applying similar regression analysis with MI (p=0.964), death (p=0.292), or repeat revascularization (p=0.069) as the dependent variables, there were no significant predictors in the model and the SS was not useful for predicting these outcomes.

## DISCUSSION

The SS was introduced as a tool to grade lesion complexity in coronary artery disease and predict clinical outcomes after PCI in patients with multivessel CAD and/or LM [1, 3]. The SS is a useful risk metric that facilitates clinical decision-making, like the most suitable revascularization method according to risk to improve clinical outcomes.

Although, the predictive value of SS in patients with multivessel and/or LM CAD is well established, its utility in other subsets of patients remains uncertain. This prompted us to investigate the utility of the SS as a predictor of MACE (angina, nonfatal MI, repeat target revascularization, cardiac death) in patients with chronic CAD (1 or 2 vessel, multivessel and/or LM disease) treated with PCI or CABG. Although the SS predicted angina in the year following revascularization, it was not predictive of MI, death or repeat revascularization.

The prognostic value of the SS has previously been investigated in patients with multivessel CAD and/or LM CAD [3-6], unprotected LM (no patent bypass graft to the left coronary artery) CAD [7-14], non-ST elevation acute coronary syndrome [16] and STEMI [17, 18]. The SS was first applied in the SYNTAX trial of 1,800 patients with multivessel and/or LM CAD. One-year and 5-year results were similar and indicated that patients with an SS >32 and between 23 and 32 were at higher risk of major adverse cardiac and cerebrovascular events (MACCE) when treated with PCI compared with those undergoing CABG [4].

 Similar results were reported in the Arterial Revascularization Therapies Study Part II (sirolimus-eluting stents for the treatment of patients with multivessel *de novo* coronary artery lesions) (ARTS II) trial, which demonstrated that the SS was an independent predictor of 5-year stent thrombosis and MACE, demonstrating its important role in risk stratification of patients with multivessel CAD [5]. In contrast, in the FREEDOM (Future Revascularization Evaluation in Patients With Diabetes Mellitus*:* Optimal Management of Multivessel Disease) trial of 1,900 diabetic patients with multivessel CAD treated with PCI or CABG, no significant interaction was observed between the revascularization strategy and the SS for 1- and 5-year clinical outcomes [6].

The prognostic value of the SS has been also investigated in patients with unprotected LM CAD treated with PCI [7-14]. The majority of these studies showed that composite ischaemic endpoints (death, MI, target lesion revascularization) were more likely in patients with higher SSs [4, 8, 10-12].

Although, the predictive value of SS in patients with multivessel and/or LM CAD is, therefore, well established, its utility in other subsets of patients remains uncertain. We have shown that SS predicted angina in the year following revascularization of patients with chronic CAD (1 or 2 vessel, multivessel and/or LM disease) treated with PCI or CABG. However, it was not predictive of MI, death or repeat revascularization.

Our study has some limitations. First, the population size was small and therefore may not have been suitably powered for the measured outcomes. The number of events is also small. This is a major limitation, but our results provide the basis for power calculations on which to base future studies. The follow-up duration was restricted to 1 year and the predictive value of the SS in this setting may change over longer timeframes.

In this patient population, the SS predicted angina in the year following revascularization but was not predictive of MI, death or repeat revascularization.

## Figures and Tables

**Table 1:** Patient demographics.

**Syntax Score Category**	**Low(<22)** **(n=83)**	**Intermediate(23-32)** **(n=36)**	**High(>32) p** **(n=21)**	p
**Age (years), *mean*** ***Minimum-Maximum*** ***95% CI***	65.8 43-97 (63.6-67.9)	70.3 46-87 (66.8-73.8)	66.9 47-83 (62.8-71.1)	0.074
**Sex (male)**	77 (92.77%)	32 (88.89%)	21 (100%)	0.291
**Smoker**	32 (38.55%)	10 (27.77%)	8 (38.1%)	0.514
**Hyperlipidemia**	47 (56.62%)	28 (77.78%)	8 (38.1%)	0.010
**Hypertension**	50 (60.24%)	30 (83.33%)	12 (57.14%)	0.034
**Diabetes mellitus**	27 (32.53%)	13 (36.11%)	6 (28.57%)	0.839
**Family History**	22 (26.5%)	7 (19.44%)	2 (9.52%)	0.222
**Overweight**	9 (10.84%)	1 (2.77%)	2 (9.52%)	0.348
**CAD**	21 (25.3%)	7 (19.44%)	4 (19.04%)	0.708

CI=confidence interval; p values refer to between-group differences; CAD= coronary artery disease.

**Table 2:** Incidence of major adverse cardiac events.

**Syntax Score Category**	**Low (<22)**	**Intermediate (23-32)**	**High (>32) **	**p**
**Angina**	8 (9.6%)	7 (19.4%)	5 (23.8%)	0.149
**MI**	1 (1.2%)	1 (2.8%)	1 (4.8%)	0.576
** New Revascularization**	4 (4.8%)	2 (5.6%)	3 (14.3%)	0.278
**Death**	5 (6.0%)	4 (11.1%)	3 (14.3%)	0.395

p values refer to between-group differences; MI=Myocardial Infarction.

**Table 3:** Logistic regression with angina as dependent variable.

	**p**	**Odds Ratio**
**Syntax**	**0.024**	**0.948**
**Total Cholesterol**	0.590	0.973
**LDL**	0.445	1.039
**TG**	0.644	1.004
**Age**	0.702	1.012
**Sex (male)**	0.999	0.000
**Smoking**	0.785	0.848
**Hyperlipidaemia**	0.263	0.496
**Hypertension**	0.821	1.162
**Diabetes Mellitus**	0.388	0.573
**Family history**	0.628	1.425
**Overweight**	0.998	0.000

LDL: low-density lipoprotein; TG: triglycerides.

## References

[1] Sianos G., Morel M.A., Kappetein A.P., Morice M.C., Colombo A., Dawkins K., van den Brand M., Van Dyck N., Russell M.E., Mohr F.W., Serruys P.W. (2005). The SYNTAX Score: An angiographic tool grading the complexity of coronary artery disease.. EuroIntervention.

[2] Serruys P.W., Onuma Y., Garg S., Sarno G., van den Brand M., Kappetein A.P., Van Dyck N., Mack M., Holmes D., Feldman T., Morice M.C., Colombo A., Bass E., Leadley K., Dawkins K.D., van Es G.A., Morel M.A., Mohr F.W. (2009). Assessment of the SYNTAX score in the Syntax study.. EuroIntervention.

[3] Serruys P.W., Morice M.C., Kappetein A.P., Colombo A., Holmes D.R., Mack M.J., Ståhle E., Feldman T.E., van den Brand M., Bass E.J., Van Dyck N., Leadley K., Dawkins K.D., Mohr F.W., SYNTAX Investigators (2009). Percutaneous coronary intervention versus coronary-artery bypass grafting for severe coronary artery disease.. N. Engl. J. Med..

[4] Mohr F.W., Morice M.C., Kappetein A.P., Feldman T.E., Ståhle E., Colombo A., Mack M.J., Holmes D.R., Morel M.A., Van Dyck N., Houle V.M., Dawkins K.D., Serruys P.W. (2013). Coronary artery bypass graft surgery versus percutaneous coronary intervention in patients with three-vessel disease and left main coronary disease: 5-year follow-up of the randomised, clinical SYNTAX trial.. Lancet.

[5] Serruys P.W., Daemen J., Morice M.C., De Bruyne B., Colombo A., Macaya C., Richardt G., Fajadet J., Hamm C., Dawkins K.D., Vranckx P., Bressers M., van Domburg R., Schuijer M., Wittebols K., Pieters M., Stoll H.P. (2008). Three-year follow-up of the ARTS-II - sirolimus-eluting stents for the treatment of patients with multivessel coronary artery disease.. EuroIntervention.

[6] Farkouh M.E., Domanski M., Sleeper L.A., Siami F.S., Dangas G., Mack M., Yang M., Cohen D.J., Rosenberg Y., Solomon S.D., Desai A.S., Gersh B.J., Magnuson E.A., Lansky A., Boineau R., Weinberger J., Ramanathan K., Sousa J.E., Rankin J., Bhargava B., Buse J., Hueb W., Smith C.R., Muratov V., Bansilal S., King S., Bertrand M., Fuster V., FREEDOM Trial Investigators (2012). Strategies for multivessel revascularization in patients with diabetes.. N. Engl. J. Med..

[7] Capodanno D., Capranzano P., Di Salvo M.E., Caggegi A., Tomasello D., Cincotta G., Miano M., Patané M., Tamburino C., Tolaro S., Patané L., Calafiore A.M., Tamburino C. (2009). Usefulness of SYNTAX score to select patients with left main coronary artery disease to be treated with coronary artery bypass graft.. JACC Cardiovasc. Interv..

[8] Capodanno D., Caggegi A., Capranzano P., Cincotta G., Miano M., Barrano G., Monaco S., Calvo F., Tamburino C. (2011). Validating the EXCEL hypothesis: A propensity score matched 3-year comparison of percutaneous coronary intervention versus coronary artery bypass graft in left main patients with SYNTAX score ≤32.. Catheter. Cardiovasc. Interv..

[9] Kim Y.H., Park D.W., Kim W.J., Lee J.Y., Yun S.C., Kang S.J., Lee S.W., Lee C.W., Park S.W., Park S.J. (2010). Validation of SYNTAX (Synergy between PCI with Taxus and Cardiac Surgery) score for prediction of outcomes after unprotected left main coronary revascularization.. JACC Cardiovasc. Interv..

[10] Morice M.C., Serruys P.W., Kappetein A.P., Feldman T.E., Ståhle E., Colombo A., Mack M.J., Holmes D.R., Torracca L., van Es G.A., Leadley K., Dawkins K.D., Mohr F. (2010). Outcomes in patients with de novo left main disease treated with either percutaneous coronary intervention using paclitaxel-eluting stents or coronary artery bypass graft treatment in the Synergy Between Percutaneous Coronary Intervention with TAXUS and Cardiac Surgery (SYNTAX) trial.. Circulation.

[11] Onuma Y., Girasis C., Piazza N. (2010). For the Interventional Cardiologists at ThoraxCenter 2000-2005. Long-term clinical results following stenting of the left main stem: insights from RESEARCH (Rapamycin-Eluting Stent Evaluated at Rotterdam Cardiology Hospital) and T-SEARCH (Taxus-Stent Evaluated at Rotterdam Cardiology Hospital) Registries.. J Am Coll Cardiol Intv.

[12] Chakravarty T., Buch M.H., Naik H., White A.J., Doctor N., Schapira J., Mirocha J.M., Fontana G., Forrester J.S., Makkar R. (2011). Predictive accuracy of SYNTAX score for predicting long-term outcomes of unprotected left main coronary artery revascularization.. Am. J. Cardiol..

[13] Shiomi H., Morimoto T., Hayano M., Furukawa Y., Nakagawa Y., Tazaki J., Imai M., Yamaji K., Tada T., Natsuaki M., Saijo S., Funakoshi S., Nagao K., Hanazawa K., Ehara N., Kadota K., Iwabuchi M., Shizuta S., Abe M., Sakata R., Okabayashi H., Hanyu M., Yamazaki F., Shimamoto M., Nishiwaki N., Imoto Y., Komiya T., Horie M., Fujiwara H., Mitsudo K., Nobuyoshi M., Kita T., Kimura T., CREDO-Kyoto PCI/CABG Registry Cohort-2 Investigators (2012). Comparison of long-term outcome after percutaneous coronary intervention versus coronary artery bypass grafting in patients with unprotected left main coronary artery disease (from the CREDO-Kyoto PCI/CABG Registry Cohort-2).. Am. J. Cardiol..

[14] Park D.W., Kim Y.H., Yun S.C., Song H.G., Ahn J.M., Oh J.H., Kim W.J., Lee J.Y., Kang S.J., Lee S.W., Lee C.W., Park S.W., Park S.J. (2011). Complexity of atherosclerotic coronary artery disease and long-term outcomes in patients with unprotected left main disease treated with drug-eluting stents or coronary artery bypass grafting.. J. Am. Coll. Cardiol..

[15] van Gaal W.J., Ponnuthurai F.A., Selvanayagam J., Testa L., Porto I., Neubauer S., Banning A.P. (2009). The Syntax score predicts peri-procedural myocardial necrosis during percutaneous coronary intervention.. Int. J. Cardiol..

[16] Palmerini T., Genereux P., Caixeta A., Cristea E., Lansky A., Mehran R., Dangas G., Lazar D., Sanchez R., Fahy M., Xu K., Stone G.W. (2011). Prognostic value of the SYNTAX score in patients with acute coronary syndromes undergoing percutaneous coronary intervention: analysis from the ACUITY (Acute Catheterization and Urgent Intervention Triage StrategY) trial.. J. Am. Coll. Cardiol..

[17] Magro M., Nauta S., Simsek C., Onuma Y., Garg S., van der Heide E., van der Giessen W.J., Boersma E., van Domburg R.T., van Geuns R.J., Serruys P.W. (2011). Value of the SYNTAX score in patients treated by primary percutaneous coronary intervention for acute ST-elevation myocardial infarction: The MI SYNTAXscore study.. Am. Heart J..

[18] Garg S., Sarno G., Serruys P.W., Rodriguez A.E., Bolognese L., Anselmi M., De Cesare N., Colangelo S., Moreno R., Gambetti S., Monti M., Bristot L., Bressers M., Garcia-Garcia H.M., Parrinello G., Campo G., Valgimigli M., STRATEGY and MULTISTRATEGY Investigators (2011). Prediction of 1-year clinical outcomes using the SYNTAX score in patients with acute ST-segment elevation myocardial infarction undergoing primary percutaneous coronary intervention: a substudy of the STRATEGY (Single High-Dose Bolus Tirofiban and Sirolimus-Eluting Stent Versus Abciximab and Bare-Metal Stent in Acute Myocardial Infarction) and MULTISTRATEGY (Multicenter Evaluation of Single High-Dose Bolus Tirofiban Versus Abciximab With Sirolimus-Eluting Stent or Bare-Metal Stent in Acute Myocardial Infarction Study) trials.. JACC Cardiovasc. Interv..

